# Refinement of the Superomedial Pedicle Technique: A New Approach to Breast Reshaping Following Reduction

**DOI:** 10.1007/s00266-023-03363-6

**Published:** 2023-05-18

**Authors:** Sara Jasionowska, Log Murugesan, Terouz Pasha, Ian C. C. King, Naveen Cavale

**Affiliations:** 1 REAL Plastic Surgery Clinic, 25 Patcham Terrace, London, SW8 4EX UK; 2 Plastic Surgery Department, Royal Free Hospital, Royal Free London NHS Foundation Trust, Pond Street, London, NW3 2QG UK; 3 Plastic Surgery Department, St Thomas’ Hospital, Guy’s and St Thomas’ Hospital NHS Foundation Trust, London, SE1 7EH UK

**Keywords:** Breast reduction, Superomedial pedicle, Breast shaping, Technique refinement

## Abstract

**Introduction:**

We present an alteration of the superomedial pedicle technique in breast reduction to control lateral fullness and create a more natural and contoured breast during reshaping. This approach has been adopted by the senior author (NC) in 79 patients over the past 4 years.

**Methods:**

A wise pattern skin incision is used, and the nipple–areola complex (NAC) is maintained on a de-epithelialized superomedial pedicle. Instead of fully releasing the pedicle from the lateral parenchyma for rotation and inset, a bridge of tissue between the pedicle on its most posterior aspect and the lateral pillar is maintained. Key holding sutures are subsequently placed in Scarpa’s fascia for reshaping.

**Results:**

We find that with this refinement, the connection with the lateral pillar pulls the lateral parenchyma medially and superiorly when the pedicle is rotated into its new position, adding a natural curve to the side. The superior medial pedicle is still attached in its postero-lateral aspect to the lateral pillar and theoretically, will provide an even more robust vascular supply to the NAC. In our series, three patients developed minor skin healing issues amenable to treatment with dressings. No one suffered from nipple loss or other serious complications, and no dog ear revisions were required.

**Conclusions:**

We present a simple alteration of the superomedial pedicle technique that we believe results in improved breast contouring. Our experience suggests that this simple adaptation is safe, effective, and reproducible.

**Level of Evidence IV:**

This journal requires that authors assign a level of evidence to each article. For a full description of these Evidence-Based Medicine ratings, please refer to the Table of Contents or the online Instructions to Authors www.springer.com/00266.

**Supplementary Information:**

The online version contains supplementary material available at 10.1007/s00266-023-03363-6.

## Introduction

Women with mammary hypertrophy or macromastia suffer from a range of physical and psychological problems [1]. To address these, several breast reduction techniques have been described [2]. The surgical principles involve the removal of excess skin and breast parenchyma without compromising nipple vascularity once it has been transposed into its new position.

The superomedial pedicle technique, used in conjunction with the Wise-pattern skin excision, has been very popular among surgeons since it was first described in the literature [3, 4]. We present a simple alteration of the superomedial pedicle to create a more projected breast with less lateral fullness and a softer and more natural contour during reshaping.

## Material and Methods—Technique Modification and Patient’s Journey

Patients are seen twice in the clinic at a minimum of two weeks before surgery. In our centre, these are done as day cases using total intravenous anaesthesia. Intraoperatively, patients are positioned supine with arms abducted on arm boards and it is the senior author’s preference not to use Infiltration to allow better visualisation and immediate control of the bleeding and to reduce the risk of subsequent haematoma. Scalpel is used instead of electrocautery to reduce local tissue thermal damage and seroma formation.

**Fig. 1:**
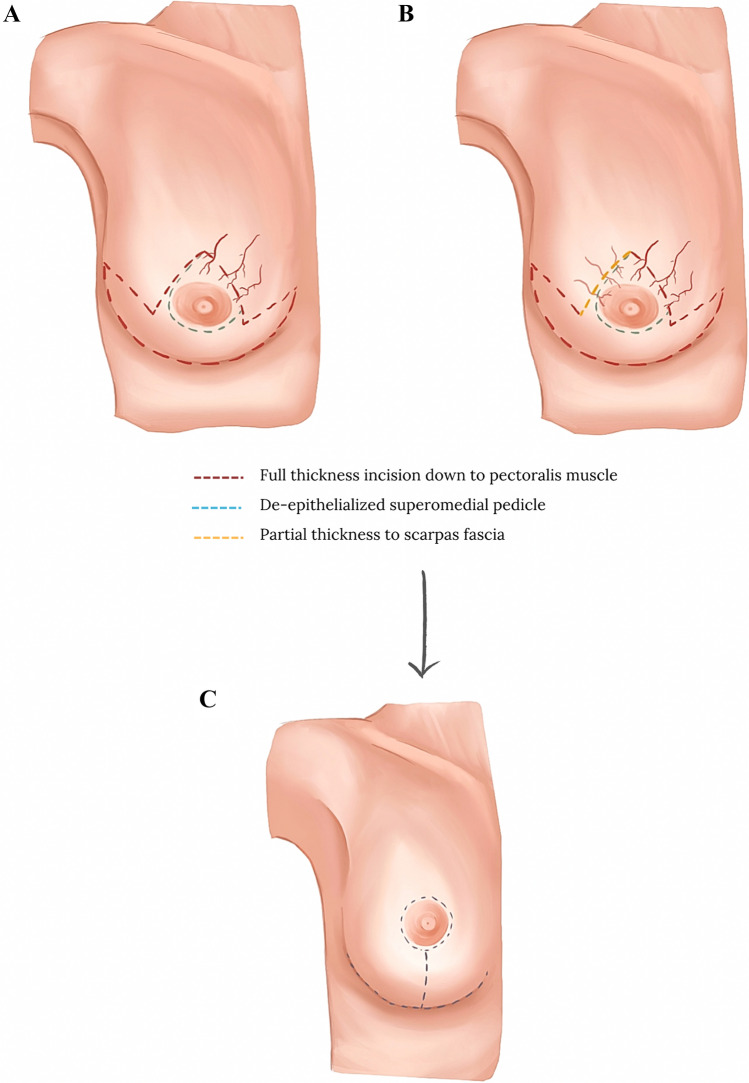
Modified superomedial pedicle incisions **A** Conventional superomedial technique, **B** Modified superomedial technique, **C** Breast reduction outcome following superomedial breast reduction (purple line—full-thickness incision down to pectoralis muscle fascia, blue line—de-epithelialized superomedial pedicle, yellow line—partial thickness incision down to Scarpa’s fascia)

Video 1 shows the nipple–areola complex (NAC) maintained on a de-epithelialized superomedial pedicle design following a Wise pattern skin excision. The medial breast parenchyma is removed followed by the excision of the lateral breast tissue. Instead of fully releasing the pedicle from the lateral pillar, a limited lateral vertical incision down to the chest wall is made, leaving a bridge of tissue between the lateral and medial tissues (Figure 1, Video 2, Video 3). The pedicle is therefore left attached to the inferior lateral breast tissue. The extent of the incision is judged intraoperatively on the arc and ease of rotation of the pedicle to accommodate for a comfortable inset (Video 4). To minimise tissue strangulation and fat necrosis, only key structural holding sutures are placed in Scarpa’s fascia along the horizontal limb, followed by a layered wound closure (2/0 vicryl for Scarpa’s fascia, 3/0 vicryl for the dermal layer, 3/0 double-ended Stratafix^TM^ for the horizontal skin limb and 4/0 monocryl for the vertical skin limb and circumareolar skin). Skin glue is applied, and wounds are dressed with 3M^TM^ Micropore tape. A bra with adjusted shoulder straps is applied to provide a snug fit to minimise swelling and for comfort. Patients are generally advised to wear a bra 24 hours a day for the first 6 weeks and are encouraged to do so for the rest of their life to reduce breast ptosis. Patients can shower the following day with the tapes on. They are offered a wound check and dressing change seven days later, and they are assessed by the operating surgeon 6 weeks after their surgery.

## Results

**Fig. 2:**
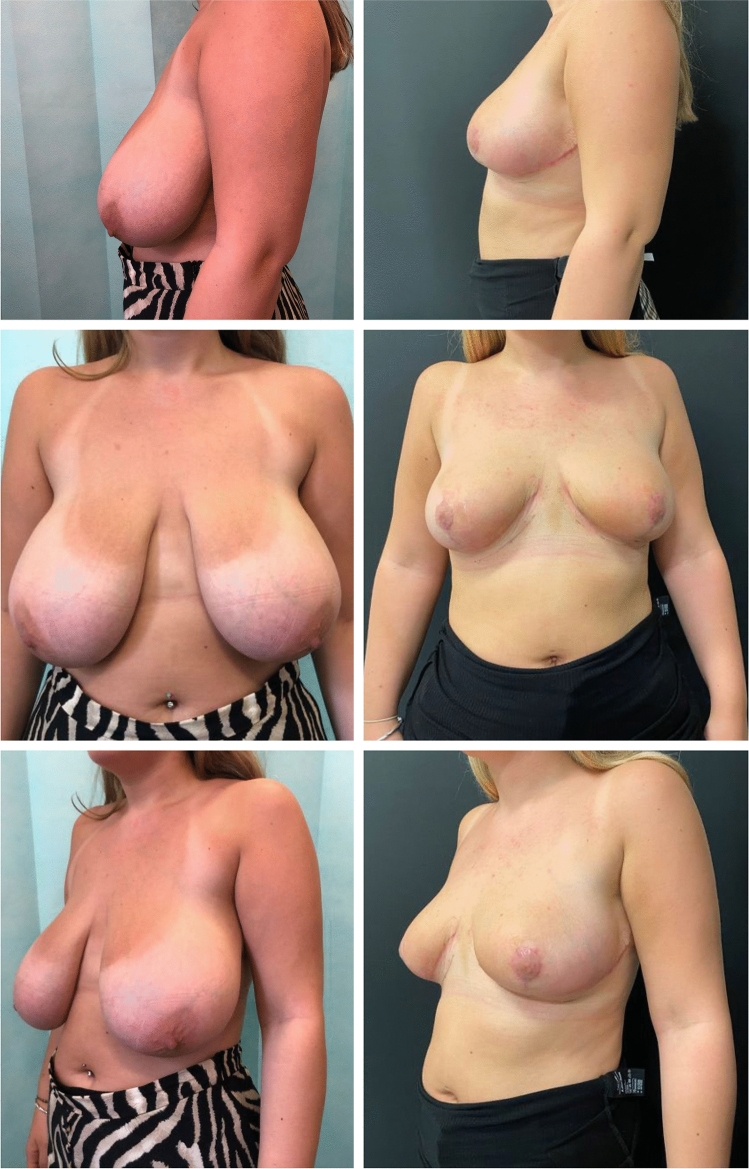
Frontal view before the surgery (top left), frontal view after the surgery (top left), left later view before the surgery (bottom left), left lateral view after the surgery (bottom left).

**Fig. 3:**
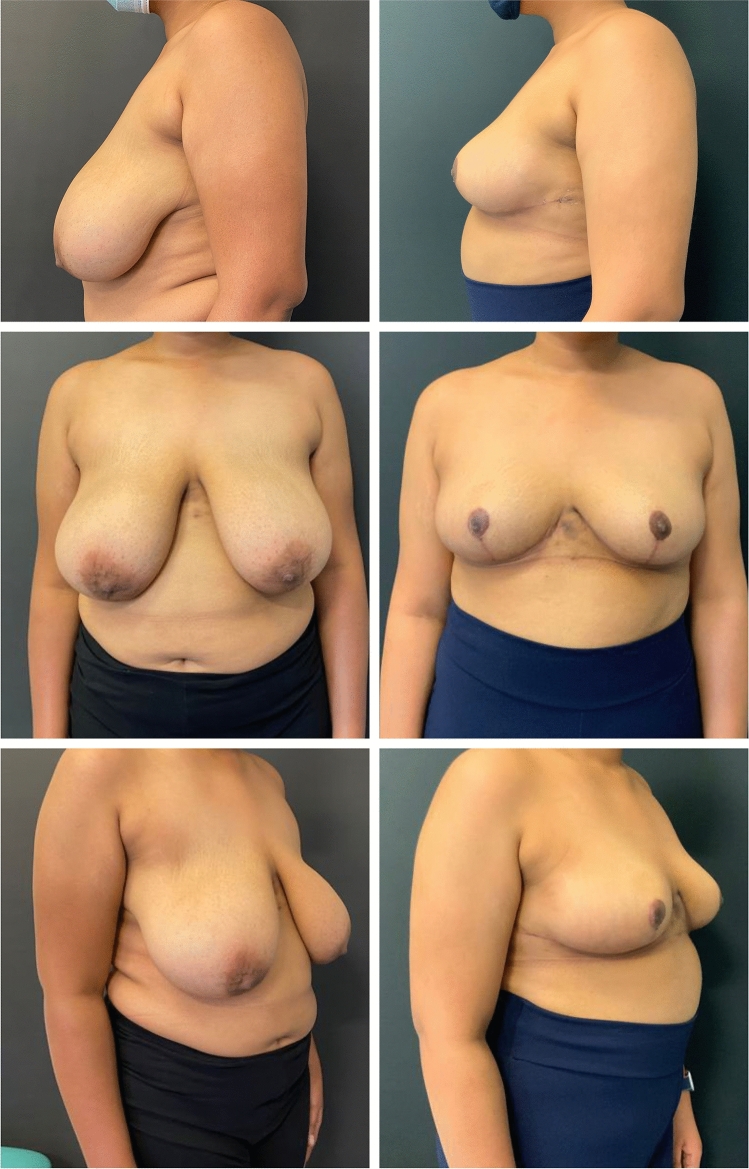
Frontal view before the surgery (top left), frontal view after the surgery (top left), left later view before the surgery (bottom left), left lateral view after the surgery (bottom left).

This method has been used in 79 patients by the senior author (NC) in the last 4 years. All patients underwent surgery for aesthetic and functional reasons. There were no major complications, no haematoma or return to theatre, no dog ear revisions, nipple loss or fat necrosis. Three patients developed mild wound healing issues which were resolved with conservative management. Patients reported high satisfaction with the aesthetic outcome. Examples of the surgical results at a 3-month follow-up are presented in Figs. 2 and 3.

## Discussion

We describe a simple modification of the superomedial pedicle technique for breast reduction. As the nipple is placed in its new position, the lateral parenchyma is pulled medially because the pedicle is still connected with the lateral pillar posterior-laterally. This reduces any potential lateral dog ear and adds a natural aesthetic curve to the lateral pole. We found this manoeuvre takes away fullness that would otherwise be noted in the more standard approach of completely releasing the pedicle from the lateral tissues. Moreover, a theoretical advantage of this modification is that the NAC's vascular base is supplemented laterally from the lateral mammary branches originating from the posterior intercostal arteries, maintaining an even more robust vascular supply to the nipple.

The inferior pedicle has previously been described as the pedicle with the most reliable vascular supply and hence, has been a very popular choice among surgeons [4]. The superomedial pedicle, originally described by Arie [5] and later refined by Orlando and Guthrie [6], is a recognised safe technique with equal or lower complication rates than the inferior pedicle. It allows for shorter operating time, less undermining and en bloc resection of breast tissue. It also provides a fuller superior breast pole, and there is less chance of bottoming out [7, 8]. The superior medial pedicle can safely be used in very large and ptotic breasts [7]. Bauermeister and colleagues [7] reported higher complication rates in patients with a nipple to sternal notch distance > 35.5 cm using the superomedial pedicle and suggested that this population should be offered a different breast reduction procedure. In our experience, when the pedicle is short (xiphisternum to clavicle distance < 25 cm) and only moved up by 3–4 cm, the pedicle does not always rotate freely, and we then fully release the pedicle from the lateral tissues. This allows for a comfortable arc of rotation and inset and therefore, a well-projected NAC. The same applies to patients with significantly large ptotic breasts. In some patients, full release allows for adjustment of the volume of the lateral pillar that some patients would like addressed as per their preferences and in proportion to their frames. The described method is not without limitations. It includes a source of bias inherent to what is subjectively perceived as enhanced contouring especially laterally. It is a relatively small sample size and is not validated by a patient outcome survey. The outcomes of this technique have not been directly compared to other breast reduction techniques.

Whilst not prescriptive, our modified approach to breast shaping using the superomedial pedicle certainly adds an additional manoeuvre that colleagues might find useful based on their intraoperative judgement.

## Conclusions

We have developed a simple alteration of the superomedial pedicle technique,which results in reduced lateral fullness and improved breast reshaping. In our experience, the technique appears to be safe, effective, and easily reproducible.

### Supplementary Information

Below is the link to the electronic supplementary material.

Video 1–De-epithelisation–superomedial pedicle design (MP4 52110 KB)

Video 2–Video showing the assembly of the superomedial pedicle before the removal of the inferior part of the breast tissue (MP4 40422 KB)

Video 3–Video showing the assembly of the superomedial pedicle and pedicle rotation after the removal of the inferior part of the breast tissue (MP4 71393 KB)

Video 4–Video showing the superomedial pedicle rotation and nipple inset (MP4 30348 KB)
